# Prevalence of dysmorphic uterus in infertile women by 3D-TVUS: a retrospective longitudinal cohort study

**DOI:** 10.1186/s12958-025-01472-2

**Published:** 2025-10-27

**Authors:** Patsama Vichinsartvichai, Sappawit Lertlerphant, Sarwinee Ratchanon

**Affiliations:** 1 LIFE by Dr. Pat, Bangkok, Thailand; 2 Department of Obstetrics and Gynecology, Faculty of Medicine Vajira Hospital, Navamindradhiraj University, Bangkok, Thailand

**Keywords:** Dysmorphic uterus, T-shaped uterus, Hysteroscopic metroplasty, 3D transvaginal ultrasound, CUME criteria, Infertility, Endometrial polyp, Septate uterus

## Abstract

**Background:**

The true prevalence and clinical significance of congenital uterine malformations (CUM), particularly the dysmorphic uterus, in infertile populations remain a matter of debate, largely due to historical limitations in diagnostic methods. This study aimed to (1) determine the prevalence of CUM in an infertile cohort using modern, objective three-dimensional transvaginal ultrasound (3D-TVUS) criteria; (2) evaluate reproductive outcomes following surgical correction; and (3) investigate associated intrauterine pathologies that may contribute to infertility.

**Methods:**

This a retrospective longitudinal cohort study included 154 women presenting for infertility investigation at a university hospital tertiary referral center between January 2020 and December 2022. All patients underwent 3D-TVUS for uterine assessment based on Congenital Uterine Malformation by Experts (CUME) criteria. Women diagnosed with CMU underwent hysteroscopic metroplasty. The primary outcomes were the prevalence of CMU subtypes and the 1-year cumulative live birth rate (LBR). Statistical analysis was performed using one-way ANOVA and chi-square tests.

**Results:**

CMU was identified in 47/154 (30.5%) women. Dysmorphic uterus was the most common anomaly, present in 42/154 women (27.3% of the total cohort; 89.4% of all anomalies). A significant association was found between dysmorphic/septate uteri and the presence of endometrial polyps (45.2%/40.0% vs. 8.5% in normal uteri; *P* < 0.001). After metroplasty, the 1-year cumulative LBR for the dysmorphic uterus group (26.2%) was comparable to the normal uterus group (20.6%; *P* = 0.907). The miscarriage rate per clinical pregnancy was also similar between the dysmorphic (15.4%) and normal uterus (25.7%) groups.

**Conclusions:**

Using modern, objective 3D-TVUS criteria, the prevalence of a dysmorphic uterus in infertile women is substantially higher than previously reported. Surgical correction may improve reproductive outcomes to levels comparable to those in infertile women with normal uterine anatomy. Furthermore, the novel association with endometrial polyps warrants further investigation and underscores the importance of a comprehensive uterine assessment during the infertility workup.

## Background

Despite significant advances in Assisted Reproductive Technologies (ART), the “uterine factor” remains a critical and often under-investigated contributor to infertility and adverse pregnancy outcomes [1, 2]. Congenital uterine malformations (CMU) affect approximately 5–7% of the general female population and up to 8–12% of infertile women [3–6]. Among women with recurrent miscarriage, the prevalence may be as high as 13–25% [3, 7]. These anomalies are associated with adverse reproductive outcomes, including recurrent pregnancy loss, preterm delivery, malpresentation, and lower live birth rates [2, 7, 8]. Thus, CMU represents not only an anatomic variation but also a clinically relevant pathology that can compromise fertility and obstetric outcomes.

This uncertainty regarding the true impact of CMU has been largely driven by the diagnostic limitations of traditional imaging methods like 2D-transvaginal ultrasound (2D-TVUS) and hysterosalpingography (HSG), which provide incomplete anatomical information [3]. The advent of three-dimensional transvaginal ultrasound (3D-TVUS) has transformed the diagnostic landscape. With high sensitivity and specificity comparable to the invasive “gold standard” of combined laparoscopy and hysteroscopy, 3D-TVUS allows for an objective, measurable, and non-invasive assessment of uterine morphology [9, 10]. This technological shift has enabled the development of standardized diagnostic criteria, such as those proposed by the Congenital Uterine Malformation by Experts (CUME) group, facilitating more precise and reliable classification of anomalies [11–13].

This enhanced diagnostic capability has led to emerging evidence suggesting that the prevalence of certain anomalies, particularly the dysmorphic (T-shaped) uterus, may be much higher in infertile populations than historical estimates indicated [4–6]. Concurrently, the long-held consensus on the surgical treatment of other anomalies, specifically the septate uterus, has been intensely debated following recent high-quality randomized controlled trials (RCTs) that question the efficacy of routine hysteroscopic metroplasty [2, 8, 14]

Therefore, the primary aims of this study were to: (1) utilize a modern, rigorous diagnostic approach combining 3D-TVUS and objective CUME criteria to establish the prevalence of CMU in a contemporary infertile population; (2) evaluate the reproductive outcomes following surgical correction of these anomalies; and (3) explore associated intrauterine pathologies that may contribute to infertility.

## Methods

### Study design and participants

This a retrospective longitudinal cohort study was conducted according to the Declaration of Helsinki for Medical Research involving Human Subjects, and the Institutional Review Board of Vajira Hospital approved the study protocol (COA 017/2564). Informed consent was obtained from all participants.

We recruited infertile women who underwent 3D-TVUS at their initial visit to the IVF unit of a university hospital, a tertiary referral center, between January 2020 and December 2022.

Inclusion criteria were women who initiated any fertility treatment cycle (intrauterine insemination [IUI] or in vitro fertilization/intracytoplasmic sperm injection), underwent surgical correction for a diagnosed CUM if applicable, and were followed for at least one year or until pregnancy was achieved. Data were retrieved from electronic medical records and digital image files. Women with other known severe causes of infertility, such as severe male factor or predicted poor ovarian response according to the Bologna criteria, were not excluded, reflecting a real-world clinical population with a high proportion of previous treatment failures. Cases with missing data were excluded from the analysis.

Participants were categorized into two groups: (1) women diagnosed with congenital uterine malformations (CMU; study group), and (2) women with a normal uterus (control group).

### Study procedures

Demographic information, obstetric history, menstrual characteristics, previous fertility treatments, and causes of infertility were extracted and recorded.

All participants underwent 3D-TVUS performed by a single experienced operator (P.V.) using a Voluson™ S10 machine with a RIC5-9 A-RS endocavity probe (General Electric Company, USA). The outer and inner contours of the uterus were evaluated in the mid-coronal plane created by the Z-technique [15, 16]. CMU was classified according to the ESHRE/ESGE recommendations [10]. For dysmorphic uterus, a diagnosis of T-shaped uterus was made if all three of the following CUME criteria were met on the 3D coronal view: a lateral indentation angle of ≤ 130°, a lateral indentation depth of ≥ 7 mm, and a T-angle of ≤ 40° [11–13].

Normal uterus controls were selected from the same infertile cohort during the same study period. A uterus was classified as “normal” if 3D-TVUS and CUME/ESHRE-ESGE morphometric criteria showed no congenital anomaly, and no structural abnormality was identified at baseline or during follow-up hysteroscopy (if performed). These women met identical inclusion and exclusion criteria and were followed using the same protocols as the CMU group.

Participants with a diagnosed CMU or suspected coexisting intrauterine pathology (e.g., submucous myoma, endometrial polyp) were scheduled for inpatient hysteroscopy. After appropriate anesthesia, diagnostic hysteroscopy was performed using a vaginoscopic technique with a 12°, 2.9 mm PANOVIEW™ Telescope (Richard Wolf GmbH, Germany). If a CMU was confirmed, operative hysteroscopy was performed. Hysteroscopic metroplasty incisions were made with the goal of visualizing both tubal ostia simultaneously from the isthmus. As an adhesion prevention measure, estradiol valerate (Progynova^®^, Bayer AG, Germany**)** 2 mg was prescribed three times daily for three weeks post-operatively [17]. A follow-up 3D-TVUS was scheduled five weeks post-procedure to confirm the anatomical result.

### Outcome measures

The primary outcomes were the prevalence of CMU subtypes and the 1-year cumulative reproductive outcomes (live birth rate, clinical pregnancy rate, miscarriage rate, ectopic pregnancy rate, and time-to-pregnancy). Outcomes in the CMU group were followed for 12 months after surgical correction, and in the normal uterus group for 12 months after the first fertility treatment cycle.

### Statistical analysis

All data were analyzed using SPSS software version 27.0 (SPSS Inc., USA). Data were presented as mean ± SD or number (%). Continuous data were compared using the independent sample t-test or one-way ANOVA. Categorical data were compared using the chi-square or Fisher’s exact test. The cumulative pregnancy rate was analyzed using Kaplan-Meier analysis with a log-rank test. A P-value of < 0.05 was considered statistically significant. No power calculation was performed as this was a retrospective analysis of all eligible patients within the study period.

## Results

A total of 154 infertile women were included in the final analysis. In total, 47 women (30.5%) were diagnosed with a CMU. The most common anomaly was a dysmorphic (T-shaped) uterus, identified in 42 women (27.3% of the total cohort). A septate uterus was diagnosed in five women (3.2% of the total cohort).

Baseline characteristics of the participants are presented in Table 1. The groups were similar in terms of age, menstrual characteristics, prior obstetric history, and other causes of infertility. A key finding was the significantly higher prevalence of endometrial polyps identified by ultrasound in participants with a dysmorphic uterus compared to those with a normal uterus (45.2% vs. 8.5%; *P* ≤ 0.001). A similarly high prevalence of polyps was observed in the septate uterus group (40.0%); however, due to the small sample size (*n* = 5), formal statistical comparisons for this subgroup are not meaningful.

**Table 1:** Baseline characteristics of participants stratified by uterine morphology

	Normal Uterus (*N* = 107)	Dysmorphic Uterus (*N* = 42)	Septate Uterus (*N* = 5)	*P*-value
Age (yr)	37.4 ± 4.1	37.8 ± 3.5	39.6 ± 3.2	0.445
Previous livebirth, n (%)	8 (7.5)	0 (0)	0 (0)	0.157
Previous miscarriage, n (%)	26 (24.2)	13 (31.0)	1 (20.0)	0.932
Had previous infertility treatment, n (%)	35 (33.0)	21 (50.0)	1 (20.0)	0.113
Total AFC	16.0 ± 3.5	16.7 ± 2.8	16.4 ± 1.5	0.563
Associated Uterine Pathologies by ultrasound				
Submucous myoma, n (%)	3 (2.8)	1 (2.4)	0 (0)	0.922
Endometrial polyps, n (%)	9 (8.5)	19 (45.2)	2 (40.0)	< 0.001

Data are presented as mean ± SD or n (%).* P*-values were calculated by one-way ANOVA or Chi-square test as appropriate. AFC; antral follicle count

As expected, the objective 3D-TVUS morphometric measurements defined by Congenital Uterine Malformation by Experts (CUME) criterias were significantly different among the three groups (Table 2). The dysmorphic and septate uteri exhibited smaller T-angles, smaller lateral indentation angles, and greater lateral indentation depths compared to normal uteri (*P* ≤ 0.001 for all comparisons).

**Table 2:** 3D-TVUS morphometric measurements by congenital uterine malformation by experts (CUME) criteria

Measurement	Normal Uterus (*N* = 107)	Dysmorphic Uterus (*N* = 42)	Septate Uterus (*N* = 5)	*P*-value
T-Angle (degrees)				
Right	62.4 ± 14.1	34.8 ± 5.2	31.5 ± 7.9	< 0.001
Left	61.9 ± 15.3	35.1 ± 4.9	30.8 ± 8.1	< 0.001
Lateral Indentation Angle (degrees)				
Right	151.3 ± 11.2	121.7 ± 9.8	112.4 ± 14.5	< 0.001
Left	152.5 ± 10.8	120.9 ± 10.1	111.9 ± 15.3	< 0.001
Lateral Indentation Depth (mm)				
Right	4.1 ± 1.8	8.6 ± 1.4	9.2 ± 1.9	< 0.001
Left	4.3 ± 1.9	8.4 ± 1.5	9.5 ± 2.1	< 0.001

Data are presented as mean ± SD*. P*-values calculated by one-way ANOVA

**Fig. 1:**
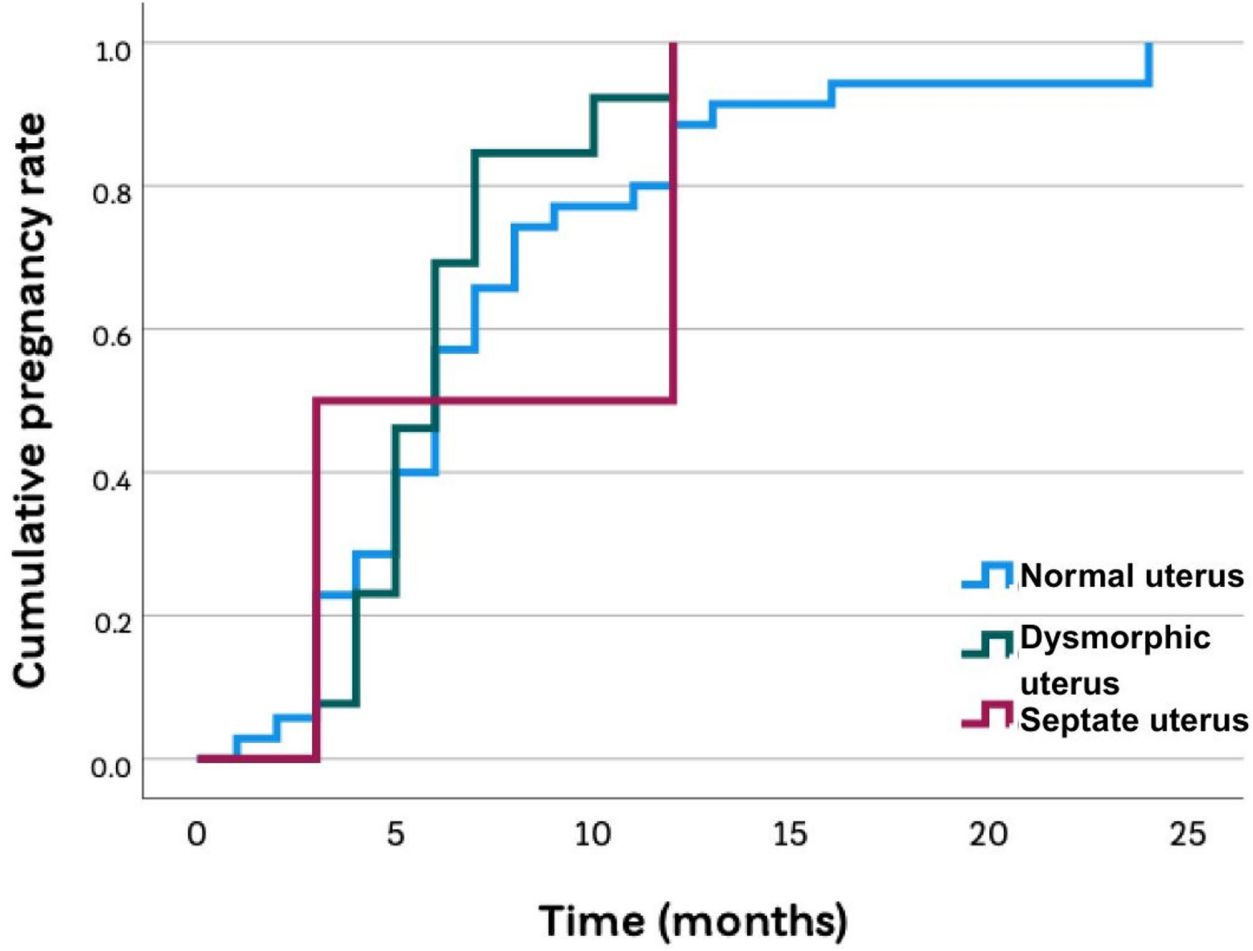
Kaplan-Meier analysis of the 1-year cumulative clinical pregnancy rate The 1-year cumulative clinical pregnancy rates among women with a normal uterus (Class U0), dysmorphic uterus (Class U1), and septate uterus (Class U2) were not significantly different (Log-rank test, *P* = 0.589)

The 1-year cumulative reproductive outcomes are presented in Table 3. After surgical correction, women with a dysmorphic uterus had reproductive outcomes comparable to infertile women with a normal uterus. There were no statistically significant differences in the primary outcome of live birth rate or other secondary outcomes between these two groups. While outcomes for the five participants with a corrected septate uterus are presented for completeness, it must be explicitly stated that this subgroup is too small to draw any valid statistical conclusions [18]. The Kaplan-Meier analysis (Fig. 1), which included all three groups, visually confirms the lack of a significant overall difference in the cumulative clinical pregnancy rate over time (*P* = 0.589).

**Table 3:** The 1-year cumulative reproductive outcomes

Outcome	Normal Uterus (*N* = 107)	Dysmorphic Uterus (*N* = 42)	Septate Uterus (*N* = 5)	*P*-value
Clinical pregnancy, n (%) [95%CI]	35 (32.7) [24.6–42.0]	13 (31.0) [19.1–46.0]	2 (40.0) [11.8–76.9]	0.916
Live birth rate (per patient), n (%) [95%CI]	22 (20.6) [14.0–29.1]	11 (26.2) [15.0–42.0]	1 (20.0) [3.6–62.4]	0.907
Ectopic pregnancy, n (%) [95%CI]	2 (1.9) [0.5–6.6]	0 (0) [0.0–8.4]	0 (0) [0.0–43.4]	0.641
Miscarriage (per clinical pregnancy), n (%) [95%CI]	9 (25.7) [14.2–42.0]	2 (15.4) [4.3–42.2]	1 (50.0) [9.5–90.5]	0.442
Time-to-pregnancy (months)	7.5 ± 5.4	6.2 ± 2.5	7.5 ± 6.4	0.685

Data are presented as mean ± SD or n (%).* P-*values was calculated by Chi-square or one-way ANOVA as appropriate

## Discussion

This study presents three principal findings: (1) a high prevalence of dysmorphic uterus (27.3%) in an infertile cohort diagnosed using objective 3D-TVUS and CUME criteria; (2) comparable 1-year reproductive outcomes after hysteroscopic metroplasty for dysmorphic uterus compared to infertile women with a normal uterus; and (3) a novel, strong association between CMU and the presence of endometrial polyps.

### Prevalence in the context of modern diagnostics

Our observed prevalence of dysmorphic uterus is substantially higher than the 1–4% reported in literature from the pre-3D ultrasound era [3, 7]. This discrepancy likely reflects a paradigm shift in diagnostics rather than a true difference in population incidence. For instance, many older studies relied on hysterosalpingography (HSG) or 2D-TVUS, methods that provide poor visualization of the external uterine contour and fundal myometrium, and could easily miss the subtle indentations characteristic of a dysmorphic uterus [10]. Our systematic application of 3D-TVUS combined with objective CUME criteria allows for precise, reproducible identification of these anatomical variations [11–13]. Furthermore, our study population’s demographics—being from a tertiary referral center enriched with patients having complex, refractory infertility and a history of treatment failure—differ from studies of general infertile populations, where a lower underlying prevalence of uterine anomalies might be expected [1, 6].

### Reproductive outcomes and the evidence for metroplasty

Following surgical correction, the live birth rate (LBR) in the dysmorphic uterus group (26.2%) was similar to that of the infertile control group. Critically, a definitive conclusion on the efficacy of metroplasty for a dysmorphic uterus is hampered by a complete absence of randomized controlled trial (RCT) evidence comparing surgery to expectant management. Therefore, our outcomes must be compared to the existing body of observational literature [19]. Our LBR is lower than the pooled LBR of approximately 56% reported in some systematic reviews of observational studies [20, 21, 22]. This difference may be attributable to the advanced mean age of our cohort, the likely more severe nature of their underlying infertility, and the relatively short 1-year follow-up period, which may not capture all eventual live births. As our study is observational, it cannot prove causality between the surgery and the outcome.

In stark contrast to the dysmorphic uterus, the debate surrounding surgical correction of the septate uterus has been directly informed by recent high-quality RCTs. Our findings in this small subgroup (*n*= 5) are insufficient for any conclusion and must be viewed in the context of this higher-level evidence. A 2024 systematic review and meta-analysis of RCTs found that septum resection did not improve the live birth rate compared to expectant management [8]. Reflecting this evolving evidence, the 2024 American Society for Reproductive Medicine (ASRM) guideline no longer recommends routine surgery, instead advocating for a shared decision-making model [2]. This highlights the critical importance of distinguishing between different CMU subtypes when considering surgical intervention.

### Novel association with endometrial polyps

Perhaps the most novel finding is the strong association between dysmorphic/septate uteri and endometrial polyps (*P* ≤ 0.001). However, this must be interpreted with caution due to a potential for detection bias. In our protocol, a CMU diagnosis often prompted a confirmatory hysteroscopy—the gold standard for polyp diagnosis—whereas controls may not have undergone hysteroscopy unless another pathology was suspected on ultrasound. This differential verification could inflate the observed prevalence of polyps in the CMU group. Nonetheless, a true biological link remains plausible. Abnormal myometrial architecture in CMU may alter uterine contractility or vascular patterns, creating an intrauterine environment conducive to polyp development, a mechanism postulated in conditions like endometriosis [23]. This finding, even with its limitations, suggests that a diagnosis of CMU on 3D-TVUS should lower the threshold for proceeding to diagnostic hysteroscopy to identify and treat potentially coexisting pathologies [24, 25, 26, 27].

### Future research directions

Looking forward, our findings underscore several critical research needs. Primarily, there is an urgent need for a well-designed, multicenter RCT to definitively establish the efficacy of hysteroscopic metroplasty for the dysmorphic uterus versus expectant management. Such a trial should include a long-term follow-up beyond one year to capture the true cumulative LBR. Additionally, the novel association with endometrial polyps requires prospective validation in a cohort where all participants undergo the same diagnostic procedures, and mechanistic studies are needed to explore potential shared pathophysiological pathways.

### Strengths and limitations

This study’s strengths include the application of modern, objective diagnostic criteria (3D-TVUS/CUME) and comprehensive follow-up within a well-defined cohort. Limitations include its retrospective, single-center design, which may restrict generalizability; the absence of a priori sample size calculation; and the critically small number of cases in the septate uterus subgroup. Furthermore, as discussed, the lack of a non-surgical control group for the dysmorphic uterus cohort precludes establishing a causal relationship between surgical intervention and outcomes.

## Conclusions

In our cohort of infertile women, the prevalence of dysmorphic uterus diagnosed by 3D-TVUS and objective CUME criteria was 27.3%, a figure substantially higher than in many historical reports. Following hysteroscopic metroplasty, women with a dysmorphic uterus achieved 1-year cumulative live birth rates comparable to infertile women with a normal uterus. Furthermore, we identified a novel, strong association between congenital uterine malformations and the presence of endometrial polyps. These findings underscore the critical importance of routine, high-resolution uterine imaging in the contemporary infertility evaluation. A definitive determination of the efficacy of surgical correction for dysmorphic uterus awaits a large-scale, randomized controlled trial.

## Data Availability

The datasets used and/or analyzed during the current study are available from the corresponding author on reasonable request.

## Abbreviations

3D-TVUS: Three-dimensional transvaginal ultrasound
AFC: Antral follicle count
ART: Assisted Reproductive Technologies
ASRM: American Society for Reproductive Medicine
CMU: Congenital uterine malformations
CUME: Congenital Uterine Malformation by Experts
ESHRE/ESGE: European Society of Human Reproduction and Embryology / European Society for Gynaecological Endoscopy
HSG: Hysterosalpingography
IUI: Intrauterine insemination
IVF: In vitro fertilization
LBR: Live birth rate
RCT: Randomized controlled trial
